# Differential Effects of In-person and Telehealth Implementation of Attachment and Biobehavioral Catch-up on Parental Sensitivity and Intrusiveness

**DOI:** 10.1007/s10995-026-04232-9

**Published:** 2026-02-07

**Authors:** Kirsten McLaughlin, Regina M. Fasano, Mary Dozier

**Affiliations:** Department of Psychological and Brain Sciences, University of Delaware, Newark, USA

**Keywords:** Telehealth, Parenting behaviors, Early intervention, Risk, Community sample

## Abstract

**Background:**

Attachment and Biobehavioral Catch-up (ABC) is a home visiting program designed for parents of children between birth and 48 months of age who have experienced early adversity. Previous research demonstrated that ABC enhances parental sensitivity and reduces parental intrusiveness through both in-person and hybrid delivery methods (Roben et al.in Child Development 88(5):1447–1452, 2017, 10.1111/cdev.12898; Schein et al. in Child Maltreatment28(1):24–33, 2023, 10.1177/10775595211072516). However, the relative effectiveness of in-person versus exclusive telehealth implementation had yet to be explored.

**Methods:**

The current study examined changes in parental sensitivity and intrusiveness from pre- to post-intervention in community implementation settings among 201 families receiving ABC either in-person or via telehealth. Parenting behaviors were analyzed through coded video recordings of parent-child free play interactions, collected before and after the intervention.

**Results:**

Parental sensitivity increased for both implementation methods, with in-person delivery showing significantly greater improvement than telehealth. Parental intrusiveness also decreased for both groups, with no significant difference between the two implementation methods.

**Conclusions:**

ABC was effective in improving parenting behaviors when delivered both in person and via telehealth in real-world community settings. Findings suggest that while telehealth delivery is a viable implementation option, in-person services may offer additional benefits for enhancing parental sensitivity, with implications for service delivery decisions in home visiting programs.

## Significance

This study examined the effectiveness of Attachment and Biobehavioral Catch-up (ABC), a program designed to enhance parental responsiveness among parents facing adversity. By comparing in-person and telehealth implementation methods, the study provides insights regarding the effectiveness of these implementation methods. This work contributes to understanding intervention accessibility for parents of infants and young children.

## Introduction

Attachment and Biobehavioral Catch-up (ABC) is a home visiting program for parents of children between birth and 48 months who have experienced early adversity, such as maltreatment (abuse/neglect), involvement in foster care or disruption in caregivers, witnessing intimate partner violence, or time spent in a neonatal intensive care unit (NICU). ABC focuses on improving parental sensitivity and decreasing parental intrusiveness, measured via a pre- and post-intervention observational parent-child free play interaction. When implemented through both in-person and a hybrid delivery approach (e.g., combining in-person visits with telehealth), ABC has demonstrated an increase in parental sensitivity and a decrease in parental intrusiveness (Roben et al., 2017; Schein et al., 2023). However, the comparative effectiveness of in-person delivery versus exclusive telehealth implementation of ABC has remained unexamined. As parents and ABC parent coaches increasingly opt for telehealth delivery post-COVID because of factors such as personal preference and the amount of time saved, it is important to assess the effectiveness of telehealth relative to in-person implementation.

### Parenting Behaviors and Child Development

Parental sensitivity and intrusiveness are critical dimensions of parenting that significantly influence child development across various domains, including emotional, social, and cognitive growth (Ainsworth et al., 1978; Feldman, 2007; Kiff et al., 2011; Murray et al., 2015; Tamis-LeMonda et al., 2004). Parental sensitivity refers to the ability of parents to accurately perceive and respond to their child’s needs and emotions in a timely and appropriate manner (Feldman, 2007; Tamis-LeMonda et al., 2004). Sensitive mothers are more likely than insensitive mothers to promote a secure attachment with their children, which fosters emotional regulation and social competence (Ainsworth et al., 1978; Cassidy, 1999). Children who experience higher levels of maternal sensitivity demonstrate better emotional adjustment and lower levels of behavioral problems than children who experience lower levels of maternal sensitivity (Feldman, 2007). Sensitive parenting is also linked with enhanced language skills and cognitive flexibility (Brody et al., 2002; Tamis-LeMonda et al., 2004). Children of sensitive parents engage in more complex play and exhibit greater curiosity and exploration than children of insensitive parents (Tamis-LeMonda et al., 2004). Furthermore, parental sensitivity during early childhood has long-term implications, contributing to academic success and positive peer relationships later in life (Brody et al., 2002).

Recent meta-analytic and longitudinal work indicates that higher levels of observed parental sensitivity are meaningfully associated with fewer child behavioral problems (Jones-Mason et al., 2023; Cooke et al., 2022; Werchan et al., 2022). Specifically, a meta-analytic review that examined 108 studies, found that parental sensitivity was significantly related to lower externalizing and internalizing symptoms across children aged 0–17 (Cooke et al., 2022). Moreover, sensitive caregiving in infancy has been shown to support the development of children’s executive functioning by promoting reward-processing systems, which in turn foster self-regulation at school entry (Werchan et al., 2022). Studies have also demonstrated that parental sensitivity moderates the influence of prenatal stress exposures on infants’ autonomic nervous system functioning, suggesting that parental sensitivity serves as a buffer, reducing physiological risk and promoting psychological resilience (Jones-Mason et al., 2023).

In contrast, parental intrusiveness is characterized by over-controlling behaviors, including excessive interference in a child’s activities or decisions (Kiff et al., 2011; Murray et al., 2015). Intrusive parenting can hinder a child’s autonomy and emotional regulation (Murray et al., 2015; Shaw et al., 2003), leading to various developmental challenges, such as increased anxiety and reduced self-esteem in children (Kiff et al., 2011). Children exposed to high levels of intrusiveness may struggle with independence and exhibit difficulties in peer interactions, as they may be less likely to develop the social skills necessary to navigate relationships than children exposed to low levels of intrusiveness (Murray et al., 2015). Children of intrusive parents often experience heightened stress responses, which can interfere with their ability to regulate emotions effectively (Shaw et al., 2003). These children may be prone to externalizing problems, such as aggression, as they struggle to manage their emotions and behaviors in social contexts (Smith et al., 2018). In fact, a recent meta-analysis of 55 studies showed that intrusive parenting was positively associated with children’s socio-emotional problems (Jiang et al., 2023).

### ABC Intervention

Given the importance of sensitive, non-intrusive parenting, parenting programs, such as ABC, have been developed to specifically focus on enhancing responsive parenting. ABC consists of ten 1-hour weekly sessions and focuses on three parenting behaviors identified as essential for children who have experienced early adversity: (1) responding to child distress with nurturance, (2) following the child’s lead with delight during everyday interactions and play, and (3) avoiding frightening behaviors (Dozier & Bernard, 2019). At each weekly session, trained ABC parent coaches discuss these targets with parents using manualized content and frequent in-the-moment comments. Manualized content includes showing example videos, citing research support, and encouraging practice activities between parents and their children. Frequent in-the-moment comments are used to support target-relevant parenting behaviors and are considered the active ingredient of ABC in promoting sensitive parental behavior (Caron et al., 2018). Randomized controlled trials (RCTs) have shown the efficacy of ABC in improving parental sensitivity and decreasing parental intrusiveness (Bick & Dozier, 2013; Yarger et al., 2020), as well as a range of child outcomes, including promoting secure attachment, self-regulation, and normalized patterns of diurnal cortisol (e.g., Bernard et al., 2012, 2015a, b; Lind et al., 2017). Across these trials, parental sensitivity has been demonstrated empirically to function as an intervention mechanism (e.g., Garnett et al., 2020; Raby et al., 2019).

### Telehealth Implementation of ABC

ABC was designed as a home visiting program, with parent coaches providing intervention sessions in parents’ homes. Parent coaches began implementing ABC via telehealth during the COVID-19 pandemic and have continued to do so in order to reach more families who cannot receive ABC in-person for various reasons (e.g., living in a remote location, sickness). Parent coaches are able to maintain frequent, high-quality in-the-moment comments while delivering ABC through telehealth (Roben et al., 2022). Parents who received telehealth implementation of ABC for some, or all, sessions showed increases in parental sensitivity and decreases in intrusive behaviors from pre- to post-intervention (Schein et al., 2023).

This aligns with a growing body of research showing that telehealth and in-person modalities produce similar outcomes. Recent meta-analyses suggest that telehealth modalities, particularly video-based services, achieve results similar to those of in-person delivery (Lin et al., 2021). One meta-analysis of randomized control trials found no significant difference in efficacy between teletherapy and face-to-face psychotherapy for depression (Lin et al., 2021). Additionally, in a large pragmatic study of behavioral-health care in rural and underserved populations, changes in depression and anxiety scores did not differ significantly between telehealth and in-person groups (Bulkes et al., 2022).

### Procedures for Training and Supervising ABC Parent Coaches

#### Screening

To qualify for training as a parent coach in ABC, candidates undergo a brief screening process conducted by ABC staff. This process utilizes two measures to identify individuals likely to implement ABC with fidelity. The first measure evaluates the candidate’s valuing of attachment and openness through the Brief Adult Attachment Interview (AAI; George et al., 1985). The second measure presents video vignettes of ABC sessions to assess the candidate’s ability to provide frequent in-the-moment comments (Caron et al., 2018). Each measure is scored on a scale of 1 to 5, with higher scores reflecting more optimal performance. Anyone, regardless of work experience or educational background, is eligible to participate in this process, allowing community members to become trained to implement ABC in their own communities.

#### Training and Supervision

After candidates pass the screening, they undergo three half-days of training that provide an overview of ABC, including manualized content and in-the-moment commenting techniques. Following this initial training, coaches engage in weekly clinical and fidelity supervision meetings for six months. Clinical supervision involves small group sessions of two to three parent coaches led by an ABC clinical supervisor, in which they review session videos and discuss case conceptualization, manual adherence, and logistical issues. Fidelity supervision consists of individual meetings with an ABC fidelity supervisor, during which supervisors select a random 5-minute clip from the parent coach’s recent session to code and review (Caron & Dozier, 2019; Meade et al., 2014). The coding of these clips is used as a tool to help ensure that parent coaches are making frequent in-the-moment comments during their sessions that meet ABC fidelity standards.

### The Current Study

Given the increase in the number of parent coaches implementing ABC through telehealth, the current study examined changes in parental sensitivity and intrusiveness from pre- to post-intervention in community implementation settings among families receiving ABC either in-person or via telehealth. We were interested in assessing whether one modality led to greater changes in parental sensitivity and parental intrusiveness than the other. Telehealth implementation of ABC was created in 2020 in response to the COVID-19 pandemic. To avoid potential confounding effects of the pandemic, study data were drawn from implementation records spanning May 2021 through early November 2023.

## Method

### Participants

A total of 776 families participated in the ABC intervention between May 1st, 2021 and November 10th, 2023. Of these families, 375 had only pre-intervention data, 70 had only post-intervention data, 130 had neither pre- nor post-intervention data, and 201 had both pre- and post-intervention data available. For the present study, the sample consisted of the 201 families who completed the ABC program and had both pre-intervention and post-intervention assessment data available as of November 10th, 2023, were included as participants. Of the 201 families, 100 received the intervention in person and 101 received the intervention via telehealth. Families were referred to parent coaches by agencies to receive ABC for various reasons (e.g., child maltreatment, involvement in foster care). The decision for families to receive ABC in person or via telehealth depended on the protocols of the referring agency.

### Agencies

The agencies employing parent coaches differed in geographic location, organizational structure, geographic reach, and the services they offered. They operated across six countries: Australia, Canada, South Korea, Sweden, the United Kingdom, and the United States. Within the United States, agencies were situated in the District of Columbia and 13 states: California, Hawaii, Kansas, Maine, Minnesota, Missouri, New York, North Carolina, Oklahoma, Pennsylvania, Utah, Virginia, and Washington. Organizationally, these agencies included nonprofits, for-profits, academic institutions, government entities, and community-based organizations. Regarding geographic reach, some focused on local or regional outreach, whereas others provided services at the state or national level. Additionally, the range of services offered varied widely, encompassing mental health and developmental support, early childhood services, counseling and therapy, and community/social support.

### Procedures

Prior to completing the first session of ABC and following the completion of the last session of ABC, families participated in a semi-structured, video recorded parent-child free play interaction. During the interaction, parents were instructed to interact with their child as they usually would using developmentally appropriate toys for about 9 min. At the pre-intervention assessment, parents also completed a demographic questionnaire. ABC parent coaches completed a survey that included questions regarding the parent’s sex and the child’s sex. The data were originally collected for program evaluation purposes and are considered to be exempt by the [redacted for blind review] Institutional Review Board.

#### Parenting Responsiveness

Parental sensitivity and intrusiveness were coded from the parent-child free play interaction on 5-point scales adapted from the National Institute of Child Health and Human Development Early Child Care Research Network’s Qualitative Scales of the Observational Record of the Caregiving Environment (Vandell, 1996). Coders rated parental sensitivity based on the extent to which the parent responded contingently to the child’s cues, with a 5 reflecting high sensitivity. Coders scored parental intrusiveness based on the extent to which the parent interfered with the child’s activity, with a 5 reflecting high intrusiveness. These scales have been used to assess parental behavior in samples with a similar age range and demographic diversity (e.g., Lind et al., 2020). Coders were trained and certified by a coding supervisor and reliability was determined by calculating the intraclass correlation coefficients (ICC) for ratings between a coding supervisor and a trained coder on a set of 10 videos. To be certified as a reliable coder, coders needed to reach agreement (an ICC of 0.80 or higher) on each of the parenting behaviors with a coding supervisor. Certified, reliable coders met weekly with a coding supervisor to review their weekly coding assignments and scores. Any discrepancies in scores were thoroughly discussed, and final scores were collectively determined by the group.

### Analytic Plan

Paired samples t-tests were first conducted to examine whether sensitivity changed from pre-intervention to post-intervention for parents who received ABC in person and for parents who received ABC via telehealth. A repeated measures Time x Implementation method analysis of variance (ANOVA) was then conducted to examine whether the implementation methods showed differential improvement from pre- to post-intervention. These steps were repeated for parental intrusiveness. All analyses were conducted using SPSS Version 6.13.

## Results

### Preliminary Analyses

Eighty-eight (44%) parents completed the voluntary demographic questionnaire at the pre-intervention assessment. Of the remaining 113 families, demographic data pertaining to the parent’s sex and the child’s sex were obtained for 28 of the families from data reported by the parent coach. This resulted in approximately 58% of participants having demographic data (*n* = 116). Descriptive statistics for these participants by implementation method are presented in Table 1. Implementation method (in-person vs. telehealth) was not significantly associated with caregiver age, caregiver sex, caregiver’s relationship to child, caregiver race, whether the caregiver identified as Hispanic, Latino, or Spanish Origin, caregiver education level, household income, child age, or child sex (all *p*-values > 0.05). Further, implementation method was not associated with pre-intervention parental sensitivity or intrusiveness (*p-*values > 0.05).

**Table 1:** Demographic statistics for participants

	Total						In Person Implementation			Telehealth Implementation			
	*N*		%	Mean (SD)	Range	*N*	%	Mean (SD)	Range	*N*	%	Mean (SD)	Range
Caregiver demographics													
Caregiver age (in years)	82			32.68 (8.30)	19.00–67.00	55		31.73 (7.12)	20.00– 55.00	27		34.73(10.12)	19.00–67.00
Caregiver sex	116					73				43			
Male	10		9			9	12			1	2		
Female	106		91			64	88			42	98		
Relationship to child	116					73				43			
Birth parent	88		76			64	88			24	56		
Foster parent/adoptive parent/kinship relation	28		24			9	12			19	44		
Caregiver race	88					57				31			
White	48		55			41	72			7	23		
Black or African American	20		23			5	9			15	48		
Asian	5		6			4	7			1	3		
Middle Eastern or Northern African	2		2			2	4			0	0		
American Indian, Alaskan Native, Indigenous, or First Nations	0		0			0	0			0	0		
Other	12		14			5	9			7	23		
Chose not to respond	1		1			0	0			1	3		
Hispanic, Latina, or Spanish origin	88					57				31			
Yes	20		23			11	19			9	29		
No	68		77			46	81			22	71		
Caregiver education level	88					58				30			
Some high school	10		11			5	9			5	17		
High school diploma or GED	22		25			18	31			4	13		
Some undergraduate level education	20		23			14	24			6	2		
Undergraduate degree	25		28			15	26			10	33		
Some master’s level education	3		3			2	3			1	3		
Master’s degree	3		3			2	3			1	3		
Some doctoral level education or a doctoral degree	3		3			0	0			3	10		
Chose not to respond	2		2			2	3			0	0		
Household income	88					58				30			
Under $15,000	14		16			8	14			6	2		
$15,000–24,999	11		13			9	16			2	7		
$25,000–34,999	8		9			3	5			5	17		
$35,000–49,999	11		13			9	16			2	7		
$50,000–74,999	15		17			10	17			5	17		
$75,000–99,999	8		9			4	7			4	13		
$100,000–149,999	12		14			8	14			4	13		
$150,000 and over	7		8			6	10			1	3		
Chose not to respond	2		2			1	2			1	3		
Child demographics													
Child age (in months)	116			20.04 (12.21)	1–48.00	73		18.42 (11.49)	1.00–48.00	43		22.79 (13.03)	5.00–48.00
Child sex	116					13				103			
Female	54		47			5	38			58	56		
Male	62		53			8	62			45	44		

### Primary Analyses

#### Parental Sensitivity

For parents who received ABC in person, a paired-samples t-test revealed a significant increase in parental sensitivity from the pre-intervention assessment (*M* = 2.54, *SD* = 0.95) to the post-intervention assessment (*M* = 3.65, *SD* = 1.05); *t*(99) = 9.08, *p* < 0.001, CI 95% [0.64, 1.09], Cohen’s *d* = 0.86). Similarly, for parents who received ABC via telehealth, a paired-samples t-test showed a significant increase in parental sensitivity from the pre-intervention assessment (*M* = 2.70, *SD* = 0.93) to the post-intervention assessment (*M* = 3.37, *SD* = 0.94); *t*(99) = 5.52, *p* < 0.001, CI 95% [0.34, 0.75]), Cohen’s *d* = 0.55).

**Fig. 1:**
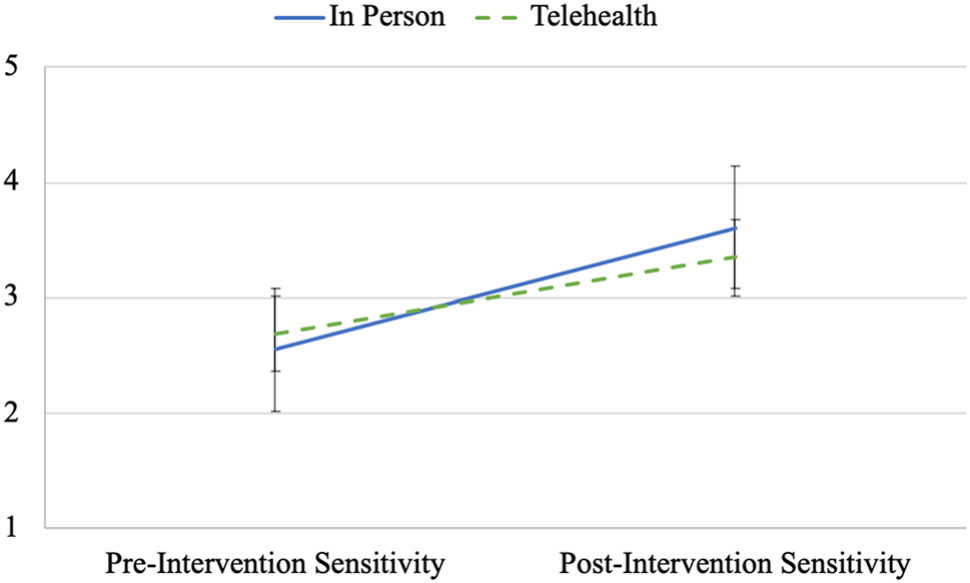
Profile plot of parental sensitivity ratings across time by implementation method. The figure above shows a profile plot for the estimated marginal means and standard errors of parental sensitivity ratings at the pre- and post-intervention assessments by implementation method

Further analyses using repeated measures ANOVA indicated a significant main effect of time on parental sensitivity (*F*(1, 199) = 108.16, *p* < 0.001, η_p_^2^ = 0.35), with scores increasing from pre-intervention to post-intervention. The main effect of implementation method was not significant (*F*(1, 199) = 0.28, *p* = 0.597, η_p_^2^ = 0.00). Of primary interest, there was a significant time by implementation method interaction on parental sensitivity (*F*(1, 199) = 6.49, *p* = 0.012, η_p_^2^ = 0.03), with a larger improvement in parental sensitivity observed for in-person implementation than for telehealth implementation. See Fig. 1.

### Parental Intrusiveness

For parents who received ABC in person, a paired-samples t-tests revealed a significant decrease in parental intrusiveness from the pre-intervention assessment (*M* = 2.17, *SD* = 1.20) to the post-intervention assessment (*M* = 1.72, *SD* = 1.02); *t*(99) = − 3.00, *p* = 0.001, CI 95% [− 0.50, − 0.11], Cohen’s *d* = − 0.31). Similarly, for parents who received ABC via telehealth, a paired-samples t-tests showed a significant decrease in parental intrusiveness from the pre-intervention assessment (*M* = 1.99, *SD* = 1.22) to the post-intervention assessment (*M* = 1.54, *SD* = 0.92); *t*(100) = − 3.80, *p* < 0.001, CI 95% [− 0.55, − 0.16], Cohen’s *d* = − 0.36).

**Fig. 2:**
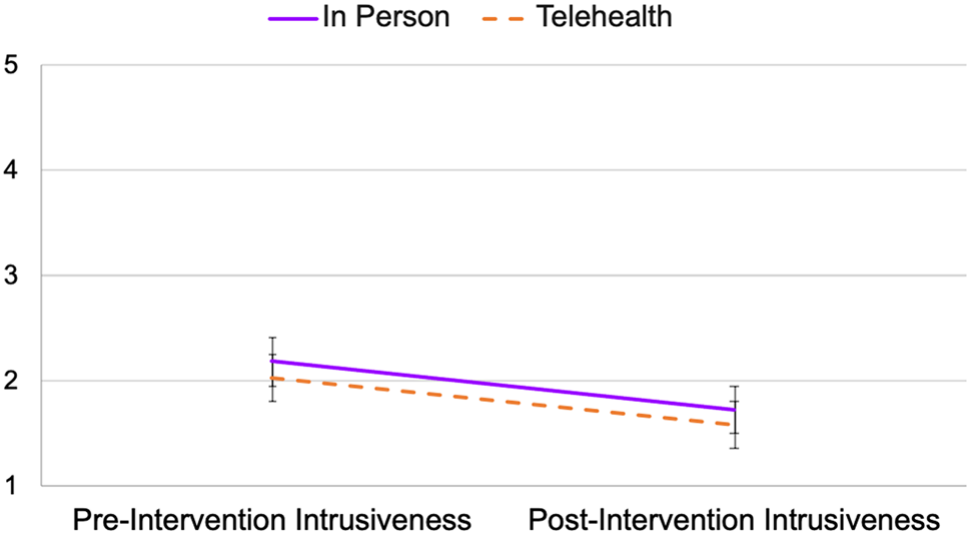
Profile plot of parental intrusiveness ratings across time by implementation method. The figure above shows a profile plot for the estimated marginal means and standard errors of parental intrusiveness ratings at the pre- and post-intervention assessments by implementation method

Repeated measures ANOVA revealed a significant main effect of time on parental intrusiveness (*F*(1, 199) = 21.23, *p* < 0.001, η_p_^2^ = 0.10), such that parental intrusiveness scores decreased from pre-intervention to post-intervention. The main effect of implementation method was not significant (*F*(1,199) = 2.24, *p* = 0.136, η_p_^2^ = 0.01), nor was the interaction effect (*F*(1,199) = 0.001, *p* = 0.982, η_p_^2^ = 0.00). See Fig. 2.

## Discussion

The current study was the first, to our knowledge, to compare changes in parenting behaviors between in-person and telehealth implementation of ABC. Findings revealed an increase in parental sensitivity and a decrease in parental intrusiveness from pre-intervention to post-intervention for both implementation methods. When directly comparing the two implementation methods, in person implementation was significantly more effective in increasing parental sensitivity than telehealth implementation, with a large effect seen for in-person and a moderate effect seen for telehealth implementation. Regarding changes in parental intrusiveness, both in-person and telehealth implementations showed small effects, with no significant difference between the two methods.

The significant increase in parental sensitivity scores from pre-intervention to post-intervention suggests that ABC enhances parental responsiveness regardless of implementation method. Notably, the larger effect size for in-person implementation compared to telehealth implementation highlights the benefits of face-to-face interaction for enhancing parental sensitivity. Despite the small effect sizes for both methods in relation to parental intrusiveness, findings still demonstrate that ABC successfully encourages a less intrusive approach to parenting when implemented in person or via telehealth. These findings are critical, as fostering autonomy in children is vital for their emotional and social development (Kochanska et al., 2001).

The finding that both implementation methods are effective at altering parental behaviors is in line with previous research on parenting programs that have shown that programs can be implemented with fidelity when delivered via telehealth (Pan et al., 2023; Traube et al., 2020). These results are promising given the cost-efficiency, ease of access, and flexibility of telehealth that allows a greater number of families to receive services. Thus, parent coaches can still implement ABC via telehealth and be confident in its efficacy in altering parental behaviors when in-person implementation is not an option. The comparative effectiveness of the two modalities—in-person implementation versus telehealth—provides important insights for the future of home-visiting parenting interventions.

### Strengths and Limitations

A strength of the study is that the study sample was diverse, including families from six countries, 13 United States, and the District of Columbia. The current study also provides further evidence of ABC’s efficacy in community settings. This is notable, as many interventions are effective in RCTs, but fail to show efficacy when implemented in community settings (Glasgow et al., 2003; Weisz et al., 1992). Although policies may have varied by geographical region, the implementation of ABC retains high fidelity across different locations due to the standardized training and fidelity supervision process for parent coaches. Furthermore, the study relied on an objective, observational measure of parenting behaviors rather than maternal reports which are subject to bias. Despite these strengths, care should be taken in interpreting these findings due to several limitations that are common in community-based treatment research.

These limitations include that there was no comparison condition or randomization of groups, nor were there measures of child outcomes. While RCTs have linked in-person implementation of ABC to a variety of positive child outcomes (Bernard et al., 2012, 2015a, b; Lind et al., 2017), more work is needed to understand the influence of telehealth implementation of ABC on these outcomes. Further, some families involved in ABC may have received other services that enhanced their parenting. Moreover, the current dataset does not include additional implementation measures that might explain why differential effects were observed for parental sensitivity but not intrusiveness. Consequently, the mechanisms underlying these modality-specific effects remain unclear and should be investigated in future research. Finally, although implementation method was determined by the agency employing the coach, if the agency allowed flexibility in delivery modality, unmeasured factors (e.g., baseline parental motivation for the ABC program, or the availability or preference of coaches for in-person delivery) may have influenced the findings. Future research should incorporate measures of these factors to better elucidate differences between in-person and telehealth delivery of ABC.

## Conclusions

The findings from this study emphasize the effectiveness of ABC in improving parenting behaviors among families facing adversity. Of primary interest, in-person implementation of ABC was shown to be more effective at increasing parental sensitivity than telehealth implementation. However, both in-person and telehealth implementations showed significant increases in parental sensitivity and decreases in parental intrusiveness, demonstrating that both approaches are effective at modifying parental behaviors. Thus, when families do not have access to in-person services, funding and resources for telehealth options can ensure that all families, particularly those in remote or underserved areas, have equitable access to these vital services. As service delivery continues to evolve, ongoing research will be vital in refining delivery methods to better meet the needs of all families and contribute to the well-being of children.

## Data Availability

The data that support the findings of this study are available from the corresponding author upon reasonable request.
